# Staphylococci, Reptiles, Amphibians, and Humans: What Are Their Relations?

**DOI:** 10.3390/pathogens13070607

**Published:** 2024-07-22

**Authors:** Valentina Virginia Ebani

**Affiliations:** 1 Department of Veterinary Sciences, University of Pisa, Viale delle Piagge 2, 56124 Pisa, Italy; valentina.virginia.ebani@unipi.it; 2 Centre for Climate Change Impact, University of Pisa, Via del Borghetto 80, 56124 Pisa, Italy

**Keywords:** amphibians, chelonians, crocodilians, lizards, snakes, *Staphylococcus*

## Abstract

Reptiles and amphibians are largely present in many environments, including domestic areas when they are kept as pet animals. They often harbor zoonotic pathogens, which can pose a serious risk of infection for humans, mainly immunocompromised individuals, the elderly, children, and pregnant women. Several studies have been carried out to verify the role of cold-blooded animals in the epidemiology of some bacteria, mainly *Salmonella*, whereas scarce attention has been focused on these animals as a source of staphylococci. These bacteria are often antimicrobial-resistant and they act as opportunistic pathogens, which can cause relevant infections in humans and animals, both domestic and wild. Asymptomatic reptiles and amphibians often harbor staphylococcal strains, such as *Staphylococcus aureus* and coagulase-negative *Staphylococcus* spp.; however, these bacteria have been associated with clinical conditions that usually appear in animals under stress conditions. In all cases, greater attention should also be focused on staphylococci in cold-blooded animals due to their implications in human and veterinary medicine.

## 1. Introduction

In the last few years, the number of reptiles and, secondarily, amphibians being kept in domestic environments as pets has significantly increased. The risk of the transmission of pathogens from these animals to their owners is high and is related to both direct contact with infected animals and indirect contact through contaminated water, fomites, and the environment [1,2]. Furthermore, transmission is possible to veterinarians, zoo personnel, and breeders who have frequent contact with cold-blooded animals that harbor pathogens. Bacteria have a relevant role in the zoonotic risk because most of them are not animal-species-specific and are able to infect humans. Studies about reptiles, and, to a lesser extent, amphibians, as sources of zoonotic bacteria mainly concern *Salmonella*, but attention has been also focused on other Enterobacteriaceae, such as *Chlamydia*, *Leptospira*, and *Mycobacterium* [2].

The members of the genus *Staphylococcus* are bacteria involved in infections of different tissue types and organs in humans and companion and farm mammals; infections in avifauna are also possible [3,4,5,6,7]. In addition, they pose a significant threat in human and veterinary medicine due to their frequent antibiotic resistance. The interest in the epidemiology of staphylococci has increased in recent years because of the emergence of some strains that have shown resistance to all available antibiotics [8]. Recent studies demonstrate that wild fauna, including mammals and birds, may be reservoirs for antimicrobial-resistant staphylococci [9,10,11]. Antimicrobial-resistant bacteria, spread through anthropogenic sources, such as industrial and domestic wastewater effluents, agricultural runoff, and garbage [12], are suspected to be the primary link to wild animals that have never undergone antibiotic treatment. Wildlife in turn can spread these agents in the environment and transmit them to other animals [12,13]. It is plausible that wild cold-blooded animals play the same role. However, little attention has been given to staphylococcal infections in reptiles and amphibians and the role of these animals in transmitting staphylococci to humans.

The aim of the present narrative review was to collect information about diseases of reptiles and amphibians associated with *Staphylococcus* spp., as well as the role of these animals as asymptomatic carriers of these bacteria, in order to better appreciate the importance of staphylococci for the health status of cold-blooded animals and for the possible risks of transmission to humans. 

## 2. Etiology

The genus *Staphylococcus* includes Gram-positive round cocci (from the Greek “kokkos”, meaning berry-like), non-spore-forming, catalase-positive, and facultative anaerobic bacteria [14]. Staphylococci are immotile and divided into several planes, forming asymmetrical grape-like clusters. The cell wall peptidoglycan is susceptible to lysostaphin and is lysozyme-resistant. The cell envelope contains ribitol-containing wall teichoic acid and membrane-anchored glycerol lipoteichoic acids [15]. Their growth temperatures range between 18 °C and 40 °C. Staphylococci have high resistance to dry conditions and high salt concentrations (10% NaCl) [15]. 

The genus, previously included in the family Micrococcaceae, is now classified as belonging to the family Staphylococcaceae. Currently, 45 staphylococcal species and 24 subspecies have been described using molecular methods; however, the accurate identification of staphylococci to the species level is quite challenging with phenotypic methods, which, in several cases, may be insufficient [16]. 

Members of the genus Staphylococcus may produce different virulence factors, including enzymes, such as coagulase, DNase, and hyaluronidase, and toxins, such as hemolysins, exfoliative toxin, enterotoxins, and toxic Shock Syndrome Toxin-1 (TSST-1) [5,17,18,19].

On the basis of their coagulase production, staphylococci are classified into two groups: coagulase-positive (CoPS) and coagulase-negative staphylococci (CoNS). In the past, CoNS were considered not pathogenic, but clinical and microbiological studies have associated several human and animal pathologies with different species of this staphylococcal group [20,21].

Although staphylococci are known commensally in the cutaneous and mucosal microflora of warm-blooded animals [15], they are frequently regarded as etiological agents of opportunistic human and animal infections. In particular, they have been identified as causes of human infections of the skin, tissue, and implants [3,7]. In addition, they represent an important concern in veterinary medicine, being involved in different clinical forms in companion and farm animals, which are often difficult to treat due to antimicrobial resistance [4,5,20].

### Staphylococcal Species in Reptiles and Amphibians

In the studies conducted on reptiles and amphibians, and reported in the following sections, *Staphylococcus aureus* and several CoNS were the strains most frequently isolated from the analyzed animals.

*Staphylococcus aureus* belongs to the CoPS group. It is the most significant human pathogenic staphylococcal species and a major cause of infection and disease in a plethora of animal hosts, significantly impacting public and veterinary health [4]. Animals can act as asymptomatic reservoirs for staphylococci transmission to humans. The prevalence of *S. aureus* in animals varies depending on the host species, including poultry, pigs, sheep, cattle, horses, and wild mammals and birds [4]. The strains of *S. aureus* isolated from companion animals are mainly of human origin and passed between human owners and their animals. Dogs and cats are not typically colonized by *S. aureus* but, rather, form transient associations that can occasionally lead to severe infections [4].

A major concern in human and veterinary medicine is related to the resistance to various antimicrobials developed by numerous *S. aureus* strains. Among them, methicillin-resistant *Staphylococcus aureus* (MRSA) has emerged and spread globally, complicating the treatment of *S. aureus* infections and creating a significant economic burden [22].

Among CoNS, *S. sciuri, S. xylosus, S. epidermidis, S. kloosii, S. capitis, S. warneri, S. arlettae*, and *S. haemolyticus* are frequently encountered in reptiles and amphibians.

*Staphylococcus sciuri* and *S. xylosus* are usually present on murine skin, and they are considered infrequent colonizers of human skin [23,24]. *Staphylococcus epidermidis* is frequently recognized on the skin and in the mucous of warm-blooded animals, including humans. Although it is considered an opportunistic agent, it is well known as one of the major causes of nosocomial infections in humans [25]. Infections have also been observed in pet and farm animals [26]. *Staphylococcus kloosii* is a rare human pathogen [27] and it is mainly found on the skin of various wild animals and only rarely on farm animals [28]. *Staphylococcus warneri* and *S. capitis* are normal inhabitants of human skin but they can cause severe infections both in humans and animals [20,29]; *S. capitis* is often isolated from the bloodstream and prosthetic joint infections and in neonatal intensive-care-unit-associated sepsis [30]. *Staphylococcus cohnii* is known as an opportunistic pathogen that can rarely cause disease in humans. It is found on farms and is sometimes involved in bovine mastitis [31]. *Staphylococcus arlettae* was first isolated from the skin and nares of poultry and goats; subsequently, it has been found in the environment, with cases of isolation from animal and human specimens reported [32]. *Staphylococcus haemolyticus* is frequently isolated from human cases of septicemia, peritonitis, otitis, and urinary tract infections [33].

## 3. Reptiles

The class Reptilia includes turtles, tortoises, snakes, lizards, and crocodiles. The Reptile Database currently contains a total of 12,162 species: 7458 Sauria, 4108 Serpentes, 365 Testudines, 27 Crocodylia, 203 Amphisbaenia, and 1 Rhynchocephalia [34].

Among reptilian diseases, cutaneous disorders are frequently diagnosed, with lizards and snakes more frequently affected than tortoises. Skin pathologies are a severe and complex issue for reptiles and usually have a multifactorial origin related to husbandry and environmental deficiencies [35].

The skin of reptiles has peculiar characteristics to protect these animals from the environment and ultraviolet irradiation and to avoid water loss. The epidermis is composed of three layers: the stratum corneum, which is heavily keratinized, and the stratum intermedium and stratum germinativum, which produce scales [35,36]. In tortoises, the stratum corneum produces the shell, consisting of the carapax and plastron; the keratinized scutes cover osteoderms that fuse with the vertebrae and sternebrae. The reptilian dermis has pigment cells, which play an important role in the animal’s color changes, especially in lizards [37]. The integumental glands, usually localized in some areas of the body and involved in reproductive and defensive behavior, influence microbiota colonization [38]. Reptiles shed their skin in regular intervals, through a process called ecdysis, related to the growth process. An exception is the scutes of the shell in tortoises, which are worn down continuously [37].

The complex structure of reptiles’ skin offers good protection to these animals, but injuries can create an access route for pathogens, such as staphylococci, which can cause infections of different severity.

Past investigations that associated staphylococci with skin pathologies were carried out using cultural methods, because molecular analyses were not available; however, bacteria identified as members of the genus *Staphylococcus* were often isolated together with other bacteria [39,40,41]. Consequently, the actual role of staphylococci in the pathogenesis of these infections is not understood, as they likely act as opportunistic pathogens as they do in mammals, and they could also be isolated due to the contamination of the analyzed specimens.

Other investigations have been carried out to study the bacterial populations in the oral cavities and/or guts of healthy animals of different reptilian species, and staphylococci have been found together with other Gram-positive and Gram-negative bacteria, suggesting that they are components of the animal microbiota in these anatomical locations.

Diseases of the reptilian gastrointestinal system include stomatitis, dental disease, regurgitation, diarrhea, colonic impaction, cloacitis, and liver disease. Stomatitis is one of the most common diseases and is related to several factors, such as stress, hypothermia, oral abrasions from caging, prey, inappropriate food items, poor sanitation, and crowding. The oral cavity in reptiles is easily traumatized, and inappropriate thermal support and inadequate sanitation can increase the bacterial colonization of wounds [42]. Staphylococci can be isolated from cases of stomatitis, even if members of the family Enterobacteriaceae, *Pseudomonas* spp., and *Aeromonas* spp. are the most frequently encountered. Similarly, staphylococci can be found in the intestines of reptiles with enteritis; however, this effect is not common in cold-blooded animals, and the observed cases have been mainly related to *Escherichia coli*, *Serratia* spp., and *Pseudomonas* spp. [42].

Staphylococci have been also found in the respiratory tracts of reptiles. They were cultured from the nasal cavities of healthy animals and from lungs affected by pneumonia. Overall, few bacterial agents have been shown to cause respiratory diseases in reptiles, and, in many cases, multiple organisms are isolated from the upper respiratory tract and lungs [43].

### 3.1. Sauria

Lasic et al. [44] studied ectodermic lesions in common wall lizards (*Podarcis muralis*); the researchers clinically analyzed 317 lizards, although they sampled cutaneous swabs from only six randomly chosen lizards for bacteriological analyses. *Staphylococcus epidermidis* was isolated from all six samples, always in association with fungi of the genus *Alternaria*. 

Recently, Strompfová and collaborators [45] analyzed skin swabs from 40 healthy captive reptiles of different orders and species living in Romania. Regarding saurians, no staphylococci were isolated from the back parts of the bodies of spiny-tailed lizards (*Uromastyx acanthinura*) and ornate mastigures (*Uromastyx ornata*), whereas *S. sciuri* was cultured from bearded dragons (*Pogona vitticeps*), *S. klosii* from leopard geckos (*Eublepharis macularius*) and western bearded anoles (*Anolis barbatus*), and *S. xylosus* from green iguanas (*Iguana iguana*) and bearded dragons.

The CoNS isolates cultured and analyzed by Strompfová et al. [45] showed high levels of resistance to several antibiotics, including ampicillin, cefoxitin, teicoplanin, and amoxicillin–clavulanic acid. Moreover, multidrug-resistant (MDR, resistant to three or more classes of antimicrobial agents [46]) isolates were often identified. In addition, the staphylococci isolated in this survey were found to be carriers of genes encoding antibiotic resistance and producers of biofilms, a factor that is pivotal for the colonization of dermal and mucosal surfaces [45].

Some studies have also investigated the presence of staphylococcal species in oropharyngeal and cloacal/fecal samples from saurians.

Tan et al. [47] detected staphylococci, including *S. aureus*, in the feces of 3/43 (6.9%) analyzed geckos (*Gecko gecko*) in Singapore. *Staphylococcus* spp. were also cultured from 21/75 (19%) fecal samples from Mediterranean house geckos (*Hemidactylus turcicus*) in Iraq, sometimes associated with *Salmonella*, *E. coli*, *Klebsiella*, and *Yersinia* [48].

In the Canary Islands (Spain), *Staphylococcus* spp. have been isolated, with high prevalence, from the feces of lizards kept in captivity; in particular, the investigated bacteria were found in all of the sampled Tenerife speckled lizards *Gallotia intermedia* and in about 90% of the La Gomera giant lizards *Gallotia bravoana* [49]. Recently, a 14.9% (11/74) prevalence of *Staphylococcus lugdunensis* was detected, for the first time in reptiles, in fecal samples from lizards of the genus *Anolis* in Tenerife (Spain) [50]. *S. lugdunensis* is considered a highly virulent CoNS species that is able to produce suppurative infections and is often antimicrobial-resistant [51]. It is usually found as a commensal on the skin and mucous membranes of humans and companion animals; therefore, it was supposed that *Anoles* may acquire these bacteria from their proximity to people and pets [50]. Although the analyzed lizards did not show evident symptoms or lesions, staphylococci were isolated from anoles with lower weight; this finding suggested that these bacteria could be involved in stomatitis, which usually causes a reluctance to feed or changes in food selection [50,52]. *Anolis* lizards are native to North America, but they have been introduced in Tenerife, as well as in numerous other areas of the world [53,54]. These lizards are an example of an invasive exotic species introduced to a new geographic area, where they can disseminate various pathogens able to affect native fauna and humans [50].

Montgomery et al. [55] isolated numerous staphylococcal species from the oral cavities of healthy captive Komodo dragons (*Varanus komodoensis*) living in Komodo National Park (Indonesia) and wild dragons of the same species captured in the same geographic area. *Staphylococcus capitis*, *S. caseolyticus* (currently classified as *Macrococcus caseolyticus*), and *S. cohnii* were isolated only from captive dragons and *S. aureus*, *S. haemolyticus*, and *S. warneri* from both wild and captive dragons; conversely, some species, such as *S. auricularis*, *S. gallinarum*, *S. hominis*, *S. kloosii*, *S. saprophyticus*, *S. sciuri*, and *S. xylosus*, were found only in wild dragons [55]. The researchers isolated other bacterial species from the examined samples as well. Overall, they found that wild Komodo dragons had both a higher average number of salivary bacteria and a much greater variety of salivary bacteria than captive dragons. These differences could be related to the diet; in particular, the routine feeding by wild dragons on putrefying carcasses [56] could be responsible for the large number of various bacteria [55].

During a recent survey over a period of three years, bacteriological analyses were carried out on specimens collected from 389 reptiles, including 125 lizards, affected by different clinical forms; only one *S. aureus* strain was isolated from the oral cavity of a *P. vitticeps* [57]. No identified CoNS species were cultured from oral and cloacal swabs collected from free-living Italian wall lizards *Podarcis sicula* and *Podarcis sicula klemmerii* in Southern Italy [58].

*Staphylococcus aureus*, *S. epidermidis*, *S. xylosus*, and *S. sciuri* were also found as part of the conjunctival flora of clinically normal captive green iguanas living in Italy by Taddei and colleagues [59].

The staphylococcal species isolated from different samples collected from lizards are reported in Table 1.

### 3.2. Testudines

Bacteriological analyses were carried out on shell biopsies collected from some tortoises (*Gopherus agassizii*) from a population living in the Colorado desert (USA) and affected by cutaneous dyskeratosis. A variety of bacteria were cultured from the shell lesions, with *Staphylococcus* and *Micrococcus* as the predominant isolates; however, in tissue sections, the organisms were confined to fissures within superficial areas of the scute. These microorganisms probably acted as secondary invaders and had little role in the development of the dyskeratosis [60].

*Staphylococcus* spp. were cultured from cutaneous swabs taken from seven adult healthy European pond turtles (*Emys orbicularis*), without visual signs of illness, living in the Silene Nature Park (Latvia); in particular, for each animal, swabs from the cloaca, feet, and mouth skin areas were analyzed and staphylococci were found in all samples [61].

Recently, Strompfová et al. [45] analyzed swabs sampled on the leg skin of some chelonians. *Staphylococcus xylosus* were cultured from marginate (*Testudo marginata*), Russian (*Testudo horsfieldii*), Greek (*Testudo graeca*), and leopard tortoises (*Stigmochelys pardalis*); *S. sciuri* was isolated from marginate, Russian, Greek, and Indian star tortoises (*Geochelonae elegans*); *S. cohnii* from Russian tortoises; *S. kloosii* from Indian star tortoises; and *S. arlettae* from leopard tortoises.

The presence of staphylococci was also investigated in cloacal swabs collected from 66 healthy red-footed tortoises (*Chelonoidis carbonaria*) at the Wild Animals Screening Center in Belo Horizonte, Minas Gerais, Brazil. Staphylococci were isolated from 48 (72.7%) animals; all isolates were CoNS: *S. sciuri* (81.3%), *S. xylosus* (12.5%), *S. kloosii* (4.2%), and *S. saprophyticus* (2%). Antimicrobial resistance was found in all strains, most of which were multidrug-resistant [62].

*Staphylococcus* spp. were cultured from cloacal and nasal swabs collected from free-ranging desert tortoises (*Gopherus agassizii*) in Arizona [63].

The conjunctival flora of captive turtles and tortoises was studied by Di Ianni and collaborators [64] in Italy. They analyzed conjunctival swabs sampled after ophthalmic examinations from 18 tortoises and 16 turtles, some of which were healthy and some had conjunctivitis. In both cases, different staphylococcal species were detected. In particular, ten *S. xylosus* isolates were cultured from tortoises (*Testudo* spp.), three of which had conjunctivitis, as well as *S. sciuri* from healthy tortoises (one *Testudo graeca*, four *Testudo hermanni*), *S. aureus* from a healthy *T. graeca*, *Staphylococcus auricularis* from a healthy *T. graeca*, *S. haemolyticus* form a turtle (*Trachemys scripta*) with conjunctivitis, and *Staphylococcus lentus* from a healthy turtle. From these findings, it is difficult to understand the role of staphylococci in conjunctivitis because they are frequently present in the ocular mucosa of chelonians. It is interesting that *S. aureus*, well known as a pathogen in warm-blooded animals, was only found in a healthy tortoise and never associated with conjunctivitis cases. Conversely, Holt and others [65] reported an *S. aureus* ocular infection in one of four *T. graeca* with panophthalmitis, although the disease was considered likely to be due to hypovitaminosis.

*Staphylococcus epidermidis* was isolated from the tympanic cavities of the middle ears of eastern box turtles (*Terrapene carolina carolina*) with aural abscesses, but also from healthy box turtles in Virginia (USA) [66]; other bacteria were isolated in terrapins with ear abscesses. Therefore, staphylococci seem to have no role in this pathology, which, otherwise, is considered to be related to hypovitaminosis A secondary to poor nutrition.

The staphylococcal species isolated from turtles and tortoises are summarized in Table 2.

*Staphylococcus* spp. have also been found in sea turtles, often as normal members of the bacterial flora in the nasal and oral cavities, gut, and conjunctiva, but they have also been cultured, usually with other bacterial species, from cases of dermatitis, stomatitis, esophagitis, gastritis, enteritis, hepatitis, pneumonia, pleuritis, pericarditis, splenitis, nephritis, conjunctivitis, and myositis [67,68].

### 3.3. Serpentes

Snakes are often affected by dermatological problems, such as the “blister disease” or “vesicular dermatitis”, in which bacteria often act as secondary pathogens and Gram-negative bacteria, mainly *Pseudomonas* spp. and *Aeromonas* spp., are most frequently involved [69]. Few data on the role of staphylococci in cutaneous disorders are available, suggesting that these bacteria are not as important in the skin lesions of snakes.

Strompfová et al. [45] isolated different staphylococcal species from the skin of the back parts of the bodies of healthy snakes; in detail, they cultured *S. kloosii*, *S. sciuri*, and *S. xylosus* from Cuban boas (*Chilabothrus angulifer*); *S. sciuri* and *S. haemolyticus* from yellow anacondas (*Eunectes notaeus*); *S. sciuri* and *S. xylosus* from common boas (*Boa constrictor*); *S. sciuri*, *S. xylosus*, *S. cohnii*, and *S. arlettae* from corn snakes (*Pantherophis guttatus*); *S. xylosus*, *S. cohnii*, and *S. warneri* from Torresian carpet pythons (*Morelia spilota variegata*); and *S. xylosus* and *S. sciuri* from Amur rat snakes (*Elaphe schrenckii*). The detection of the same staphylococcal species found by these researchers during the same survey involving snakes, lizards, and turtles shows that there is not a correlation between bacterial species and reptilian species and suggests that animals acquire the staphylococcal species that are present in the environment.

Hilf et al. [70] isolated CoNS from the upper airways of healthy common boas, Indian pythons (*Python molurus*), and reticulated pythons (*Python reticulatus*) kept in the Pittsburg Zoo (Pennsylvania, USA); CoNS were also cultured from the lungs of two *P. molurus* that died from pneumonia [70]. More recently, *S. sciuri* was isolated from farm edible king rat snakes (*Elaphe carinata*) and Oriental rat snakes (*Ptyas mucosus*) with both pneumonia and enteritis [71].

The presence of staphylococci in the oral cavity has been investigated in different snake species. Goldstein et al. [72] analyzed the oral cavities of captive garter snakes (*Thamnophis* spp.), both adult and newborn, in New York State (USA); CoNS were the most common isolated bacteria from both groups of snakes. The same researchers also performed bacteriological analyses on venom sampled from healthy, captive Southern Pacific rattlesnakes (*Crotalus viridis helleri*) and Mojave rattlesnakes (*Crotalus scutulatus scutulatus*); *S. aureus* and CoNS were isolated [73].

Blaylock et al. [74] cultured *S. epidermidis* from the oral cavities of healthy snakes of different species in Southern Africa, and Fitzgerald et al. [75] isolated other CoNS species from oral swabs collected from prairie rattlesnakes (*Crotalus viridis*) in Colorado, USA.

The oral microbiota of healthy captive snakes living in Brazil was investigated by Fonseca et al. [76], who detected Gram-negative and Gram-positive bacteria, including staphylococci. In particular, they isolated CoNS from South American rattlesnakes (*Crotalus durissus*), green anacondas (*Eunectes murinus*), and swamp racers (*Mastigodryas bifossatus*) and *S. aureus* from short-tailed coral snakes (*Micrurus frontalis*).

*Staphylococcus lentus*, *S. xylosus*, and no-typed *Staphylococus* spp. have been isolated from the oral and cloacal swabs of healthy Burmese pythons (*Python molurus bivittatus*) transported from Anh Thien’s farm, located in Ho Chi Minh, Vietnam, to a private zoo in Korea [77].

Oral swabs from 12 common lanceheads (*Bothrops atrox*) with stomatitis, bred in a Brazilian farm for venom production, were submitted to bacteriological analyses. *Staphylococcus* spp. were isolated from 26.31% of the animals; other bacterial species, both Gram-positive and Gram-negative, were also isolated. Thus, in these snakes, staphylococci also seem to act as opportunistic agents [78].

The bacterial oral flora of snakes, including venomous ones, has attracted great interest with regard to understanding the potential risk of infection for humans, such as breeders and veterinarians, who often are attacked. In fact, it has been observed that individuals rarely develop bacterial infections of wounds after a snakebite [79].

Some authors [73] have suggested that venom is sterile and the bacteria, such as staphylococci, present in venom samples are those present in the oral cavity. However, it has been suggested that infections after a snakebite are rare because of the antimicrobial activity of snake venom.

The most recent studies have identified different components with antibacterial properties, including activity against staphylococci, in the venom of different snake species, such as the jararaka (*Bothrops jararaca*) [80], whitetail lancehead (*Bothrops leucurus*) [81], Marajó lancehead (*Bothrops marajoensis*) [82], crossed pit viper (*Bothrops alternatus*) [83], Eastern diamondback rattlesnake (*Crotalus adamanteus*) [84], rattlesnake (*C. durissus cumanensis*) [85], and rainforest hognosed pit viper (*Porthidium nasutum*) [86]. Recently, Khan et al. [87] demonstrated that the venom of both the puff adder (*Bitis arietans*) and the Samar spitting cobra (*Naja samarensis*) possessed anti-biofilm activity against methicillin-susceptible *S. aureus* and methicillin-resistant *S. aureus*. Furthermore, the venom of the puff adder, Egyptian cobra (*Naja haje*), and red spitting cobra (*Naja pallida*) was tested for antimicrobial activity by Okumu et al. [88] and was found to be active against *S. aureus*.

The staphylococcal species encountered in different ophidian species are summarized in Table 3.

### 3.4. Crocodylia

Staphylococci have rarely been isolated from diseased crocodilians. The first case was reported by Mainster et al. [89], who cultured *S. aureus* from several American alligators (*Alligator mississippiensis*) exhibiting a respiratory disease [89]. Several years later, *S. cohnii* was cultured from the blood of one American alligator that had tan-colored friable lesions on the jaw and dorsal skin [90]. Moreover, staphylococci, as well other bacteria, have been associated with some cases of crocodilian septicemia [91].

Overall, crocodilians are considered resistant to several infections, probably due to their high levels of serum antimicrobial activity [92]. Staphylococci, as in warm-blooded animals, can act as opportunistic pathogens also in crocodilians, which, under stress conditions, may develop diseases with different degrees of severity.

During a study on fresh water crocodiles (*Crocodylus siamensis*), an antibacterial compound from the blood was partially purified and functionally characterized and given the name ‘crocosin’, which exhibited activity against salmonellae and *S. aureus*; it has been speculated that this compound may be used as a defense mechanism towards bacterial infections in freshwater [93].

Staphylococci have been isolated from healthy crocodilians too. *Staphylococcus epidermidis* was found as part of the intestinal flora of wild Nile crocodiles (*Crocodylus niloticus*) in the Okavango Delta, Botswana [94], and Silva et al. [95] found that *Staphylococcus* spp. were present in 14.4% of the bacterial isolates in the oral cavities and cloacae of Broad-snouted caimans (*Caiman latirostris*) born and bred in captivity in a zoo in Paraíba, Brazil. Similarly, *S. aureus*, *S. hyicus*, and other non-specified *Staphylococcus* species have been found in the oral cavities of wild American crocodiles (*Crocodylus acutus*) and Morelet’s crocodiles (*Crocodylus moreletii*) captured in the Mexican Caribbean [96].

Staphylococci have been also isolated from farmed crocodilian meat, probably as a result of the contamination that occurs during slaughter and dressing procedures [97]. Farmed crocodilians, bred for meat and skin commercialization, may pose a severe threat to personnel working with them or their products. Moreover, the transmission of bacteria from crocodilians to humans can occur through injuries [98], frequent in personnel working with these animals, and during activities in crocodile waters [99].

The staphylococcal species isolated from crocodilians are shown in Table 4.

## 4. Amphibians

Currently, 8737 amphibian species are recognized: 222 caecilians, 816 salamanders, and 7699 frogs [100]. Free-living amphibians are largely distributed in different environments worldwide. Frogs are also farmed for meat production, appreciated for its soft texture, mild flavor, and nutritional properties [20]. In addition, some amphibian species are kept in captivity as pets [101].

Data about staphylococcal infections in amphibians are very limited. These bacteria have been associated, together with other Gram-positive and Gram-negative bacteria, with “red leg syndrome” [102]. This is a systemic bacterial disease, often lethal, associated with cutaneous erythema, which occurs most often on the ventrum or extremities and is due to vasodilation, congestion, and petechial, paintbrush, or ecchymotic hemorrhages. Animals affected by red leg syndrome may also show anorexia, swelling, coelomic effusions, and epidermal alterations, such as erosions, ulcers, sloughing, or necrosis [103,104].

Staphylococci have been isolated from the skin of amphibians, in which they act as opportunistic pathogens. Recently, *Staphylococcus* spp., mainly *S. sciuri*, were found as components of the skin and gut microbiome of caecilians (*Herpele squalostoma*) in Cameroon (Central Africa) [105].

Conversely, the pathogenic role of staphylococci in cutaneous diseases has not been fully understood. This also because it is well documented that amphibian skin is rich in various antimicrobial peptides [106], which could interfere with bacterial infections.

Interesting insights about the susceptibility of frogs to *S. aureus* infection were presented by Pickof in a past study (1923) [107]. He found that common laboratory frogs (reported by the author as *Rana vernalis*) experimentally infected with a strain of *S. aureus*, virulent to humans, did not develop disease. Pickof reported two well-defined mechanisms in frogs for the destruction of staphylococci. The first mechanism occurs when the inoculated bacteria pass readily into the general circulation; in this case, the Kupffer cells of the liver exhibit marked phagocytic activity, and, in a few hours, almost all bacteria that were circulating in the bloodstream are destroyed. The second mechanism occurs when the anatomic conditions prevent the early passage of the inoculated bacteria into the general circulation; in this case, the bacteria are phagocytosed by leukocytes as a local defense mechanism [107].

Although *Staphylococcus* spp. seem not to cause relevant diseases in amphibians, these animals harboring staphylococci could be a source of bacteria for humans, as documented by Slaughter and collaborators [108]. These researchers studied the staphylococcal population in Cope’s gray treefrogs (*Hyla chrysoscelis*) in East-Central Kansas (USA), where these amphibians are routinely used in elementary and secondary science classrooms. In particular, fecal samples were collected over a 3-week period from 12 Cope’s gray treefrogs and analyzed. The frogs were collected in the field, transported to the laboratory, and housed in individual containers at room temperature in a facility free from other animals. A total of 222 CoNS strains were isolated; the predominant species encountered was *S. sciuri* (50%), followed by *S. xylosu*s (43%), six *S. felis* (3%), four *S. cohnii* (2%), three *S. gallinarum* (1%), one *S. saprophyticus* (0.5%), and one *S. capitis* (0.5%). The species’ distribution among the isolated staphylococci was similar in all frogs over the entire sampling period. This finding, besides being related to the conditions in which the frogs were kept, suggests that the staphylococcal species isolated were related to the initial environment. Most of the isolates were resistant to antibiotics, mainly penicillin [108].

Furthermore, contaminated frog meat can be a source of staphylococci for consumers, as shown by investigations on the main edible species [109], including *Ptychadena mascareniensis* and *Ptychadena pumilio* [110], *Hoplobatrachus occipitalis* [111], and *Lithobates catesbeianus* [112]. Bacteria are killed by the cooking process, but individuals may contract staphylococci during raw meat manipulation.

The staphylococcal species isolated from amphibians are summarized in Table 5.

## 5. Discussion

The studies carried out on reptiles and amphibians show that staphylococci act as opportunistic bacteria in these animals, similarly to their actions in mammals and birds. In most cases, various CoNS species have been isolated from specimens collected from both healthy and sick cold-blooded animals. *Staphylococcus aureus* has been frequently cultured as well. Reptiles and amphibians infected by staphylococci develop disease when they are under stress due to concomitant infections and improper living conditions.

Some studies demonstrate that *Staphylococcus* strains isolated from reptiles and amphibians are often resistant to one or more antimicrobials. This is a current and relevant concern regarding their implications in veterinary and human medicine. In fact, antimicrobial-resistant staphylococcal strains, including methicillin-resistant staphylococci, may pose a direct problem for infected cold-blooded animals that need a specific therapy, as well as for other animals and humans that have contact with infected reptiles and amphibians or with an environment contaminated by them.

A further issue related to staphylococci is their ability to transfer genetic material to other bacteria; in fact, members of the genus *Staphylococcus* can act as donors of genes coding for virulence factors, such as biofilm production, and antimicrobial resistance, contributing to an increase the pathogenic activity of other bacterial strains [113,114].

Cold-blooded animals can be source of staphylococci for humans in different manners. Frequently, they are kept as pets in domestic environments, where they represent a risk of infection, mainly for immunocompromised individuals, the elderly, children, and pregnant women. Similarly, they can be a source of bacteria for domestic animals living in the same house [2].

Breeders of reptiles and amphibians are at high risk of transmission because of their frequent contact with the animals, which, in addition, can sometimes attack and injure them [2,98].

A particular threat is related to the manipulation of meat from animals, such as frogs and crocodiles, bred for this purpose and harboring zoonotic pathogens. The proper processing and cooking of frog and reptile meat usually destroys these organisms, but improper handling and cooking may lead to food-borne infections. Usually, the attention is focused on meat as a source of *Salmonella*, but other bacteria, including *Staphylococcus* spp., can be present [97] and should be investigated.

Conversely, cold-blooded animals mainly acquire staphylococci from the environment, including water, where they live, and, for gut bacteria, from the diet [115]. The major mechanism behind the acquisition of bacteria for some reptiles is through close contact with their mothers shortly after hatching; studies indicate that lizard mothers may transfer microbes to their progeny vertically in different ways depending on their biology [50,116], but it is possible that this also happens in other reptiles. Similarly, caecilians acquire staphylococci and other bacteria by eating the mother’s skin cells; extended skin-to-skin contact with neonates and the nutritional provisioning of offspring likely promote the vertical transmission of skin and gut microbiomes, as found in Congo caecilians (*H. squalostoma*) [105]. In contrast, minimal vertical transfer, due to occasional physical contact between the parent and their eggs or offspring, occurs in several frog and salamander species [105].

Additionally, it cannot be excluded that reptiles and amphibians acquire staphylococci from humans, mainly through manipulation by breeders, owners, and veterinarians. In fact, no staphylococcal species specific to cold-blooded animals are known, and those isolated from specimens collected from these animals are the same usually encountered in humans, mainly on the skin and in mucous membranes [50].

## 6. Conclusions

Reptiles and amphibians may be a source of not only salmonellae and other pathogens but also of commensal bacteria, which can cause different issues.

The pathogenic capacity of staphylococci for reptiles and amphibians is currently unknown. However, it seems that staphylococci act as opportunistic agents also in cold-blooded animals. In addition, the role of staphylococci in reptiles and amphibians is not easy to understand, because their isolation is often due to sample contamination. The staphylococcal species isolated from reptiles and amphibians are the same encountered in other animals and humans; therefore, it is possible that they acquire staphylococci mainly from their environments and diets. Their life conditions, such as in captivity or in the wild, may influence the staphylococcal populations harbored by reptiles and amphibians.

Further studies should be carried out on these animals to better identify the staphylococcal species involved in their pathologies, as well as those present in clinically healthy animals. Studies to determine the antimicrobial resistance characteristics of *Staphylococcus* isolates are pivotal to increase the knowledge about this current issue, particularly from a One Health perspective.

## Figures and Tables

**Table 1:** Staphylococcal species isolated from lizards.

Animal Species	Location	Sample	Health Status	*Staphylococcus* Species	Ref.
*Anolis barbutus*	Slovakia	skin	healthy	*S. kloosii*	[45]
*Anolis* spp.	Tenerife (Spain)	feces	lower weight	*S. lugdunensis*	[50]
*Eublepharis macularis*	Slovakia	skin	healthy	*S. kloosii*	[45]
*Gallotia bravoana*	Tenerife (Spain)	feces	healthy	*Staphylococcus* spp.	[49]
*Gallotia intermedia*	Tenerife (Spain)	feces	healthy	*Staphylococcus* spp.	[49]
*Gecko gecko*	Singapore	feces	healthy	*S. aureus*	[47]
*Hemidactylus turcicus*	Iraq	feces	healthy	*Staphylococcus* spp.	[48]
*Iguana iguana*	Slovakia	skin	healthy	*S. xylosus*	[45]
	Italy	conjunctiva	healthy	*S. aureus*, *S. epidermidis*, *S. sciuri*, *S. xylosus*	[59]
*Podarcis muralis*	Serbia	skin	ectodermic lesions	*S. epidermidis*	[44]
*Podarcis sicula*	Italy	oral, cloacal swabs	healthy	*Staphylococcus* spp. CoNS	[58]
*Podarcis sicula klemmerii*	Italy	oral, cloacal swabs	healthy	*Staphylococcus* spp. CoNS	[58]
*Pogona vitticeps*	Slovakia	skin	healthy	*S. xylosus*, *S.sciuri*	[45]
	Romania	oral cavity	-	*S. aureus*	[57]
*Varanus komodoensis*	Indonesia	oral cavity	healthy	*S. aureus*, *S. auricularis*, *S. capitis*, *S. caseolytocus*, *S. cohnii*, *S. gallinarum*, *S. haemoliticus*, *S. hominis*, *S. kloosii*, *S. saprophyticus*, *S. sciuri*, *S. xylosus*, *S. warneri*	[55]

**Table 2:** Staphylococcal species isolated from chelonians.

Animal Species	Location	Sample	Health Status	*Staphylococcus* Species	Ref.
*Chelonoidis carbonaria*	Brazil	cloaca	healthy	*S. kloosii*, *S. sciuri*, *S. saprophyticus*, *S. xylosus*	[62]
*Emys orbicularis*	Latvia	skin	healthy	*Staphylococcus* spp.	[61]
*Geochelonae elegans*	Slovakia	skin	healthy	*S. kloosii*, *S. sciuri*	[45]
*Gopherus agassizii*	Colorado	skin	cutaneous dyskeratosis	*Staphylococcus* spp.	[60]
	Arizona	cloaca, nasal cavity	healthy	*Staphylococcus* spp.	[63]
	Italy	conjunctiva	healthy	*S. xylosus*	[64]
	Italy	conjunctiva	conjunctivitis	*S. xylosus*	[64]
*Stigmochelys pardalis*	Slovakia	skin	healthy	*S. arlettae*, *S. xylosus*	[45]
*Terrapene carolina carolina*	Virginia (USA)	ear	ear abscess	*S. epidermidis*	[66]
	Virginia (USA)	ear	healthy	*S. epidermidis*	[66]
*Testudo graeca*	Slovakia	skin	healthy	*S. sciuri*, *S. xylosus*	[45]
	Italy	conjunctiva	healthy	*S. aureus*, *S. auricolaris*, *S. sciuri*, *S. xylosus*	[64]
	Italy	conjunctiva	conjunctivitis	*S. xylosus*	[64]
	United Kingdom	conjunctiva	panophthalmitis	*S. aureus*	[65]
*Testudo hermanni*	Italy	conjunctiva	healthy	*S. sciuri*, *S. xylosus*	[64]
	Italy	conjunctiva	conjunctivitis	*S. xylosus*	[64]
*Testudo horsfieldii*	Slovakia	skin	healthy	*S. cohnii*, *S. sciuri*, *S. xylosus*	[45]
	Italy	conjunctiva	conjunctivitis	*S. xylosus*	[64]
*Testudo marginata*	Slovakia	skin	healthy	*S. sciuri*, *S. xylosus*	[45]
	Italy	conjunctiva	healthy	*S. xylosus*	[64]
*Trachemys scripta*	Italy	conjunctiva	healthy	*S. lentus*	[64]
	Italy	conjunctiva	conjunctivitis	*S. haemolyticus*	[64]

**Table 3:** Staphylococcal species isolated from snakes.

Animal Species	Location	Sample	Health Status	*Staphylococcus* Species	Ref.
*Boa constrictor*	Slovakia	skin	healthy	*S. sciuri*, *S. xylosus*	[45]
	Pennsylvania (USA)	upper respiratory tract	healthy	CoNS	[70]
*Bothrops atrox*	Brazil	oral cavity	stomatitis	*Staphylococcus* spp.	[78]
*Chilabothrus angulifer*	Slovakia	skin	healthy	*S. kloosii*, *S. sciuri*, *S. xylosus*	[45]
*Crotalus durissus*	Brazil	oral cavity	healthy	CoNS	[76]
*Crotalus scutulatus* *scutulatus*	New York, USA	oral cavity	healthy	*S. aureus*, CoNS	[73]
*Crotalus viridis*	Colorado (USA)	oral cavity	healthy	CoNS	[75]
*Crotalus viridis helleri*	New York (USA)	oral cavity	healthy	*S. aureus*, CoNS	[73]
*Elaphe carinata*	China	-	pneumonia, enteritis	*S. sciuri*	[71]
*Elaphe schrenckii*	Slovakia	skin	healthy	*S. sciuri*, *S. xylosus*	[45]
*Eunectes muremis*	Brazil	oral cavity	healthy	CoNS	[76]
*Eunectes notaeus*	Slovakia	skin	healthy	*S.sciuri*, *S. haemolyticus*	[45]
*Mastigodryas bifossatus*	Brazil	oral cavity	healthy	CoNS	[76]
*Micrurus frontalis*	Brazil	oral cavity	healthy	*S. aureus*	[76]
*Morelia spilota variegata*	Slovakia	skin	healthy	*S. cohnii*, *S. xylosus*, *S. warneri*	[45]
*Pantherophis guttatus*	Slovakia	skin	healthy	*S. arlettae*, *S. cohnii*, *S. sciuri*, *S. xylous*	[45]
*Ptyas mucosus*	China	-	pneumonia, enteritis	*S. sciuri*	[71]
*Python molurus*	Pennsylvania (USA)	upper respiratory tract	healthy	CoNS	[70]
	Pennsylvania (USA)	lung	pneumonia	CoNS	[70]
*Python molurus bivittatus*	Korea	oral cavity, cloaca	healthy	*Staphylococcus* spp.	[77]
*Python reticulatus*	Pennsylvania (USA)	upper respiratory tract	healthy	CoNS	[70]
*Thamnophis* spp.	New York (USA)	oral cavity	healthy	CoNS	[72]

**Table 4:** Staphylococcal species isolated from crocodilians.

Animal Species	Location	Sample	Health Status	*Staphylococcus* Species	Ref.
*Alligator mississipiensis*	Florida, USA	lung	respiratory disease	*S. aureus*	[89]
	Florida, USA	blood	skin lesion	*S. cohnii*	[90]
*Caiman latirostris*	Brazil	oral cavity, feces	healthy	*Staphylococcus* spp.	[95]
*Crocodylus acutus*	Mexico	oral cavity	healthy	*S. aureus*, *S. hyicus*, *Staphylococcus* spp.	[96]
*Crocodylus moreletii*	Mexico	oral cavity	healthy	*S. aureus*, *S. hyicus*, *Staphylococcus* spp.	[96]
*Crocodylus niloticus*	Botswana	feces	healthy	*S. epidermidis*	[94]

**Table 5:** Staphylococcal species isolated from amphibians.

Animal Species	Location	Sample	Health Status	*Staphylococcus* Species	Ref.
*Herpele squalostoma*	Cameroon	skin, gut	healthy	*S. sciuri*, *Staphylococcus* spp.	[105]
*Hoplobatrachus* *occipitalis*	Nigeria	meat	healthy	*Staphylococcu* spp.	[111]
*Hyla chrysoscelis*	Kansas (USA)	feces	healthy	*S. capitis*, *S. cohnii*, *S. felis*, *S. gallinarum*, *S. saprophyticus*, *S. xylosus*	[108]
*Lithobates* *catesbeianus*	Brazil	meat	healthy	*Staphylococcus* spp.	[112]
*Ptychadena* *mascareniensis*	Nigeria	meat	healthy	*Staphylococcus* spp.	[110]
*Ptychadena pumilio*	Nigeria	meat	healthy	*Staphylococcus* spp.	[110]
